# Japanese conservative messages propagate to moderate users better than their liberal counterparts on Twitter

**DOI:** 10.1038/s41598-021-98349-2

**Published:** 2021-10-04

**Authors:** Mitsuo Yoshida, Takeshi Sakaki, Tetsuro Kobayashi, Fujio Toriumi

**Affiliations:** 1 Department of Computer Science and Engineering, Toyohashi University of Technology, Aichi, Japan; 2 Institute for Future Initiatives, The University of Tokyo, Tokyo, Japan; 3 Department of Media and Communication, City University of Hong Kong, Kowloon Tong, Hong Kong; 4 School of Engineering, The University of Tokyo, Tokyo, Japan

**Keywords:** Computational science, Scientific data, Human behaviour, Complex networks

## Abstract

To examine conservative–liberal differences in the extent to which partisan tweets reach less partisan moderate users in a nonwestern context, we analyzed a network of retweets about former Japanese Prime Minister Shinzo Abe. The analyses consistently demonstrated that partisan tweets originating from the conservative cluster reach a wider range of moderate users than those from the liberal cluster. Network analyses revealed that while the conservative and the liberal clusters’ internal structures were similar, the conservative cluster reciprocated the follows from moderate accounts at a higher rate than the liberal cluster. In addition, moderate accounts reciprocated the conservative cluster’s following at a higher rate than they did for the liberal cluster. The analysis of tweet content showed no difference in the frequency of hashtag use between conservatives and liberals, but there were differences in the use of emotion words and linguistic expressions. In particular, emotion words related to the propagation of messages, such as those expressing “dislike”, were used more frequently by conservatives, while the use of adjectives by conservatives was closer to that of moderate users, indicating that conservative tweets are more palatable for moderate users than liberal tweets.

## Introduction

A growing body of research indicates fragmentation of political communication on social media^1^. Not only do people tend to consume politically homogeneous information on social media selectively, but they also tend to “friend” politically like-minded others selectively and disassociate from others with incongruent views^2^. As a result, politically cross-cutting communication on social media decreases and communication becomes fragmented^3,4^. Political communication within homogeneous echo chambers becomes polarized through the reinforcement of prior attitudes, and negative feelings toward political outgroups lead to affective polarization.

However, politics is only one topic on social media, and many people do not show great interest or involvement^5^. While political fragmentation and polarization on social media are often reported, the percentage of people posting and sharing politically active and partisan messages is relatively small. If highly partisan social media users only internally share and circulate partisan messages within their own closed echo chambers for mutual affirmation, they will rarely reach the majority of nonpartisan, ideologically moderate users. Put differently, if partisan echo chambers are closed structures and partisan messages do not spill over to persuadable moderate users, then the impact of fragmentation and polarization on social media on public opinion will be limited.

However, in reality, partisan messages spill over from fragmented clusters, reaching less partisan people with less interest in politics. While some argue that the cost of “liking” or retweeting partisan messages is low, so the real political consequences of such “clicktivism” are small^6^, others argue that when a message attracts a large number of “likes” and retweets, the partisan messages become visible on social media and can have an impact on public opinion, including those who are not politically active^7^. In addition, clicktivism allows partisan messages to attract many “likes” and retweets, which draws the attention of the media, political elites, and celebrities^8^. As political elites respond to partisan messages and the media reports on related social media movements, partisan messages reach even politically inactive people and raise their awareness, ultimately impacting political behavior such as voting^9–11^. Therefore, in examining the impact of political communication on social media, it is necessary to assess not only the level of fragmentation and polarization, but also the extent to which partisan messages spilling over from fragmented partisan echo chambers reach the apolitical and moderate mainstream. Against this backdrop, this study examines three research questions about Twitter. (a) To what extent do partisan tweets reach moderate users beyond partisan clusters? (b) Are there are differences in the degree of reach between ideological conservatives and liberals? (c) If there are conservative–liberal differences, what causes them?

By focusing on Twitter, we deal with the public’s individualized and spontaneous political expression, rather than the organized mobilization and persuasion by political parties and established political groups. This means that we are concerned with connective action that is “individualized and technologically organized sets of processes that result in action without the requirement of collective identity framing or the levels of organizational resources”^12^. According to Bennett and Segerberg^12^, “[t]he linchpin of connective action is the formative element of ‘sharing’: the personalization that leads actions and content to be distributed widely across social networks”, which is entirely in line with the scope of this study which analyzes retweets on Twitter.

Previous studies in Western democracies show that the nature and form of clicktivism differ between conservative and liberal people. Liberals in the American Twittersphere tend to focus on online activism using hashtags, while conservatives tend to create their own information ecosystem with its own partisan media because they have little trust in the mainstream media^8^. As a result, fragmented users who only retweet messages from their in-group partisan media are more likely to be conservative than liberal. This is consistent with the finding that conservatives have more homogeneous networks on Twitter than liberals^13^. On the other hand, liberal messages are more likely to be covered by the mainstream media; therefore, they are more likely to reach a wider range of ideological individuals and institutions than conservative messages.

However, it is debatable whether conservative Twitter users are more disconnected from moderate users than liberal users. For example, the probability of coming across cross-cutting content on Facebook has been found to be higher for conservatives than for liberals^3^, suggesting that conservative clusters may have more networks extending beyond their own cluster, which is not necessarily consistent with the finding that conservatives have more homogeneous networks than liberals. One potential reason for these inconsistencies in the findings is the context of research. Because political institutions and culture influence political communication on social media, it is crucial to examine it in various political contexts to obtain findings with high generalizability. Nevertheless, the propagation of partisan messages has rarely been studied in contexts other than the United States and a few Western democracies. Therefore, this study examines the aforementioned research questions by probing Twitter use in Japan, the democracy with the longest history in Asia.

Following the definition of ideology as the “total structure of the mind”^14^, we define ideology as the complex network of values, attitudes, and beliefs that, taken as a whole, constitute an interpretive framework through which to view the world. The liberal (progressive) versus conservative contrast described by Mannheim applies to the current ideological landscape of Japan. Ideology in Japan primarily revolves around foreign policy and national security, and “policy based party competition in Japan is inherently one dimensional—no matter which substantive dimension of policy is considered, the parties are ranked in essentially the same way”^15^.

Conservatives in Japan have spent a long time shaping its partisan discourse and refining its narratives in a way that is palatable even to less partisan people^16^. Liberals, on the other hand, have a long history of organized offline activities based on labor unions and have not adapted effectively to online activism. In the early days of the Internet in Japan, there was a temporary “liberal bias” because users were skewed toward the urban and highly educated segments^17^. However, this gap has now disappeared, and conservative parties such as the incumbent Liberal Democratic Party have adapted better to the online environment^18^. This is consistent with the argument, illustrated by European right-wing parties, that those with a vertical organizational structure are more efficient in online mobilization and represent their constituents more effectively^19^. This difference in the efficiency of social media outreach between conservative and liberal (or right- and left-wing) parties may be seen not only in mobilization messages originating from the political elites, but also among general users. That is to say, in the context of Japan, it is plausible that conservatives are more efficient in reaching politically inactive moderate users than liberals. Based on these theoretical arguments, this study aims to describe the difference between Japanese conservatives and liberals in the degree to which retweeted messages originating from each partisan cluster can reach politically inactive moderate users on Twitter, as well as the causes of this difference.

## Method

We analyzed the retweet network concerning former Prime Minister Shinzo Abe. In order to examine the liberal–conservative asymmetry in reaching out to the middle, it is necessary to limit the analysis to political tweets in some way. While many Japanese are apolitical and nonpartisan^20^, the prime minister is the most important political actor and receives a lot of media attention, making it easy for even apolitical people to hold attitudes of liking or disliking the prime minister. Moreover, since Japanese people use their likes and dislikes of the prime minister as a cue to determine their policy attitudes^21^, the extent to which positive and negative messages about the prime minister reach politically inactive moderate users plays a vital role in determining their policy attitudes. For these reasons, we limited my analysis to tweets about Prime Minister Abe.

From Twitter API, a total of 129,639,061 tweets were collected from February 10, 2019 to October 7, 2020 that included the word “Abe”. The word “Abe” could be expressed either as Chinese characters (“安倍”) or Japanese Katakana (“アベ”). The Japanese Katakana version of “Abe” was included because opponents of the Abe administration often use it when referring critically to the former Prime Minister. The Abe administration ended on September 16, 2020, but because many retrospective evaluations of the Abe administration’s performance and of former Prime Minister Abe himself were posted, tweets dated up to October 7 were collected to cover these evaluations.

Next, we divided the collected tweets into monthly segments and clustered the tweets for each month. Specifically, we clustered the top 500 retweets for each month using the method of Uchida et al.^22^. Bae et al. reported that 70% of the tweets retweeted more than 100 times had a retweet duration of ten days or less^23^. This finding means that we need to set the time window to more than ten days to include popular tweets retweeted for a more extended period in our analysis. Therefore, we set it to one month. In our data, 41% of the tweets retweeted more than 100 times lasted for more than ten days, which indicates that we can include the long-circulating tweets in our analyses. In constructing the tweet network, we applied weights based on the similarity of each link, namely the Simpson coefficient. We define the Simpson coefficient as follows:

$$\mathrm{Simpson}\,(A, B)=\frac{|A\cap B|}{\mathrm{min}\{\left|A\right|, \left|B\right|\}}$$

For community detection, we employed the Louvain method, one of the modularity-based community detection methods, representing the degree of connectivity between a set of clusters^24^. By maximizing modularity, the Louvain method detects clusters with high connectivity. More specifically, it quickly clusters the network by performing local optimization and aggregating nodes that belong to the same community. Existing studies show that the Louvain method performs better than other modularity optimization methods such as Clauset, Newman, and Moore^25^, Pons and Latapy^26^, and Wakita and Tsurumi^27^.

We then listed the Twitter accounts that retweeted the tweets for each cluster each month. We mapped the clusters across months by treating clusters with a high agreement in the list of accounts with those in adjacent months as politically homogeneous and stable. Specifically, The similarity between cluster C_*M,p*_ in month M and cluster C_*M*+*1,q*_ in month M + 1 is defined as.

$${\text{sim}}\left( {{\text{C}}_{M,p} ,{\text{C}}_{M + 1q} } \right) = {\text{N}}\left( {{\text{C}}_{Mp} \cap {\text{C}}_{M + 1q} } \right)/{\text{N}}\left( {{\text{C}}_{M + 1q} } \right),$$

where N(C) is the number of users who have retweeted the tweets in cluster C at least once. Among all the cluster combinations between adjacent months, if the similarity is maximum and greater than 0.2, the two clusters are considered to be the same cluster. As a result, two clusters emerged that remained stable throughout the data collection period. After manually examining the content of the tweets in each of these two large clusters, we determined that the clusters consisted of ideologically conservative tweets (N = 651) and liberal tweets (N = 3,001) (see SI1 in the Supplementary Information online for the sample tweets).

From the detected conservative and liberal clusters of tweets, “core accounts” with strong partisanship were defined. Specifically, an account was defined as a core liberal account if it retweeted tweets from the liberal cluster more than 10 times and retweeted more than twice as many tweets from the conservative cluster. Similarly, we defined an account as a core conservative account if it retweeted the tweets of the conservative cluster more than 10 times and retweeted more than twice as many tweets as those of the liberal cluster. The cut-off of 10 times was set as a criterion to include at least 70% of the accounts that retweeted tweets from the liberal/conservative cluster at least once. With this criterion, 94% of the accounts that retweeted tweets from the liberal cluster at least once and 78% of the accounts that retweeted tweets from the conservative cluster at least once were defined as core accounts. The 10 retweets criterion is commonly found in previous studies^28,29^. As a result, 134,855 core liberal accounts and 88,709 core conservative accounts were identified. Users who did not belong to either the liberal or conservative core accounts were defined as “noncore accounts”, which are supposed to be less partisan, moderate users. Of the tweets from the liberal cluster, 91.1% were posted by core liberal accounts. Of those from the conservative cluster, 62.2% were posted by core conservative accounts.

### Analysis

**Figure 1:**
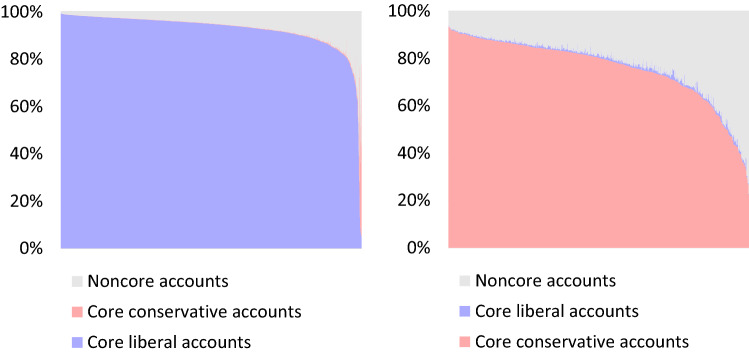
Reach of core conservative/liberal tweets to noncore accounts.

First, we analyzed whether tweets from the conservative cluster or the liberal cluster are more likely to reach noncore accounts. For this purpose, we calculated the following “noncore rate” to see how many tweets from the conservative and liberal clusters are retweeted by noncore accounts.

$$\mathrm{Noncore\,rate}=\frac{1}{N}\sum \frac{\# of\,noncore\,users}{\# of\,All\,RT\,users},$$

where N is the number of tweets in each cluster. The higher the noncore rate, the more likely retweets by noncore accounts are; i.e., the more likely a tweet is to be shared and seen by more moderate users in addition to highly partisan users. The noncore rate for tweets belonging to the liberal cluster was 6.46%, and for the conservative cluster it was 23.23%. The difference between the two ratios was statistically significant (*t*_(726)_ = 31.29, *p* < 0.001 (two-tailed), Cohen’s *d* = 1.60). In other words, conservative tweets have greater reach to less partisan moderate accounts than liberal tweets. To show this difference visually, we plotted the number of retweets on the horizontal axis (tweets on the left have more retweets), and the ratio of core liberal accounts, core conservative accounts, and noncore accounts among the retweeters is shown on the vertical axis. Figure 1 shows the plot of liberal tweets on the left and that of conservative tweets on the right.

As shown in Fig. 1, more than 80% of the top 95.4% of liberal tweets are retweeted by liberal core accounts. In contrast, only the top 50.7% of conservative tweets have more than 80% of their retweets from conservative core accounts. There is a pronounced asymmetry in that liberal tweets are overwhelmingly retweeted by core liberal accounts, while noncore accounts retweet a substantial portion of conservative tweets. Therefore, we can conclude that in Japan, partisan tweets about Prime Minister Abe from the conservative side are more likely to reach moderate users than those from the liberal side. What causes this liberal–conservative asymmetry in reaching out to the middle? First, we tested the network hypothesis that conservative and liberal users differ in their reach to less partisan, moderate users because of differences in the structure of the networks they each constitute on Twitter. The retweet network and the follow relationships serve different analytical purposes. The retweet network is suitable for analyzing the propagation of messages. Therefore, we first analyzed the retweet network of tweets from the conservative and liberal clusters to understand the difference in reaching moderate users. On the other hand, connections among accounts represent more stable relationships of users. Therefore, in the following analyses, we analyzed the connections among accounts to probe why tweets from the conservative cluster propagate better than those from the liberal cluster.

### Network hypothesis

**Figure 2:**
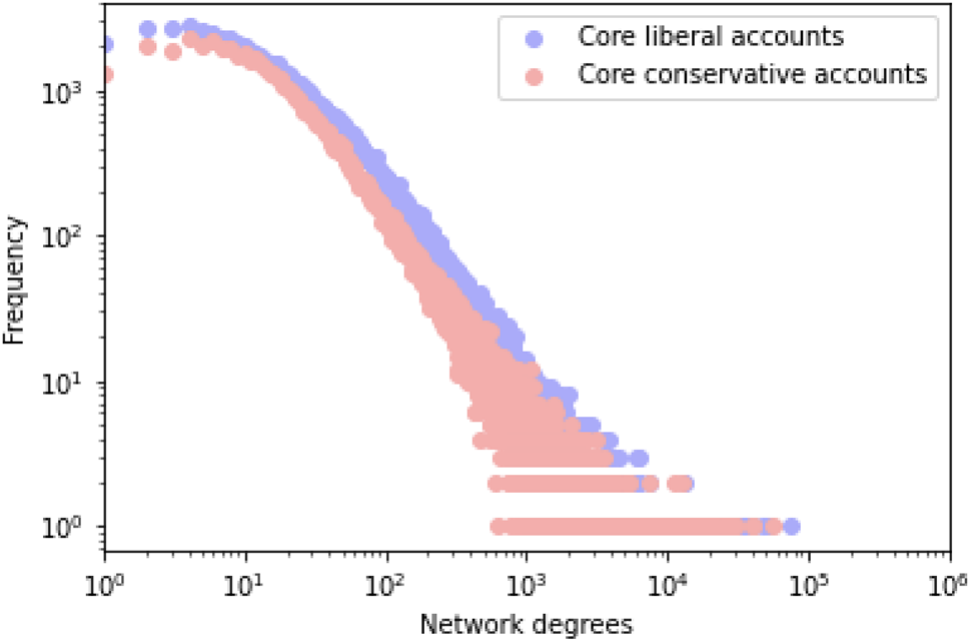
Distributions of network degrees inside partisan clusters.

First, the extent of reach to moderate users could be attributed to a difference in network structure within the two clusters. Specifically, suppose the network density of the core liberal cluster is higher than that of the core conservative cluster; liberal tweets would be more likely to be retweeted and circulated inside the cluster, making them less likely to spill over beyond the cluster. Figure 2 compares the distributions of the network degrees between core liberal accounts and core conservative accounts. Each of these two networks is an undirected graph created with the core liberal and core conservative accounts as nodes and each following relationship with the account as an edge. As shown in Fig. 2, the two distributions are similar, indicating no difference between liberals and conservatives in terms of the network structure inside the clusters.

**Figure 3:**
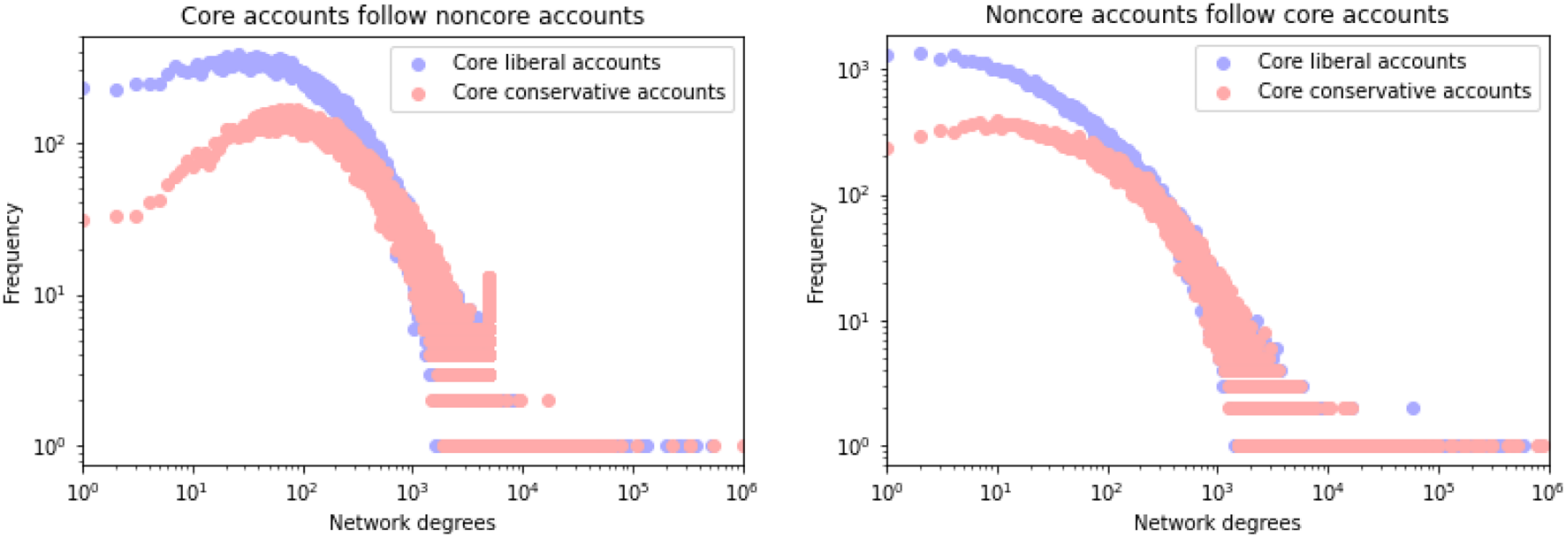
Degrees of edges between core and noncore accounts.

Next, we examine how partisan users are connected to moderate users by analyzing the network structure between core and noncore accounts. This is a network in which one end of an edge is a core account while the other is a noncore account, which reflects the openness of conservative and liberal cluster structures. The left-hand side of Fig. 3 shows the distribution of the degrees of edges in which core accounts follow noncore accounts, while the right-hand side shows that of noncore accounts following core accounts. In both figures, the distributions of core conservative networks are shifted to a higher order than those of core liberal accounts, indicating better connections to noncore accounts. On the left side of Fig. 3, the mean degree was 681.7, and the median 300 for the liberal cluster, while the mean degree was 940.4 and the median 450 for the conservative cluster. On the right-hand side of Fig. 3, the mean degree was 654.4 and the median 152 for the liberal cluster, while the mean and median were 732.4 and 253, respectively, for the conservative cluster. These results indicate that core conservative accounts have a more outwardly open network structure than core liberal accounts.

To examine the networking differences between core liberal and core conservative accounts further, we analyzed the reciprocity of followership. Because bidirectional linkage is less likely to be disconnected than unidirectional linkage^30^, the follow-back rate at least partially reflects the strength of conscious motivation to build and maintain a network that extends beyond the partisan clusters. We calculated the percentage of noncore accounts that follow core accounts *and* are followed back by liberal and conservative core accounts. On average, the follow-back rate by the liberal cluster of noncore accounts is 51.0%, whereas for conservative clusters it is 61.8%, which is a highly significant statistical difference (*t*_(179774)_ = –90.26, *p* < 0.001 (two-tailed), Cohen’s *d* = 0.40).

We next examined the extent to which liberal and conservative core accounts are followed back by noncore accounts. This indicates the extent to which core accounts’ follows are reciprocated when they try to expand their reach by following less partisan, moderate users. This indicator is important because messages from partisan clusters will not reach moderate accounts if the core accounts are not followed back when they follow noncore accounts. Among the noncore accounts followed by core accounts, 29.9% of liberal and 38.0% of conservative core accounts are followed back, which is a statistically significant difference (*t*_(169391)_ = –70.36, *p* < 0.001 (two-tailed), Cohen’s *d* = 0.32). Therefore, core conservative accounts enjoy higher reciprocity of followership from noncore accounts.

### Contents hypothesis

So far, the results support the network hypothesis that conservatives have greater reach to moderate users than liberals because of better structural connections to moderate users. However, it is not only the difference in network structures that leads to asymmetric reach to moderate users. It is also possible that differences in tweet content creates a difference in their acceptance by moderate users. For example, liberals may be more likely to use uncivil and aggressive criticism of conservative Prime Minister Abe, which may cause them to be shunned by moderate users, resulting in lower reach. To examine these differences in the tweet content, we analyzed (a) the use of hashtags, (b) the use of emotion words, and (c) the similarity of linguistic expressions between moderate users.

Hashtags make it easier for politically like-minded people to share tweets on a particular issue^8^. At the same time, hashtags may prevent partisan messages from spilling over beyond clusters by facilitating the circulation of the messages only within the partisan clusters. For example, #安倍やめろ (“Step down, Abe”) was a common hashtag used by liberals who were critical of the Prime Minister. While these hashtags make it easier for highly partisan liberals to find political in-group members and boost their solidarity, their strongly partisan implications may make moderate users hesitate to retweet them. This is because noncore users are less interested in politics and less partisan, so they are less likely to share tweets containing hashtags reflecting intense partisanship. Therefore, the use of partisan hashtags may restrict the propagation of partisan tweets to within the cluster and suppress their reach to noncore users. Among the liberal cluster tweets, 9.3% used hashtags, compared with 12.0% of the conservative cluster tweets. These ratios were statistically indistinguishable (χ^2^_(1)_ = 3.561, *n.s.*). Therefore, the hypothesis that liberal tweets are less likely to reach noncore users because they use partisan hashtags more frequently was not supported.

**Figure 4:**
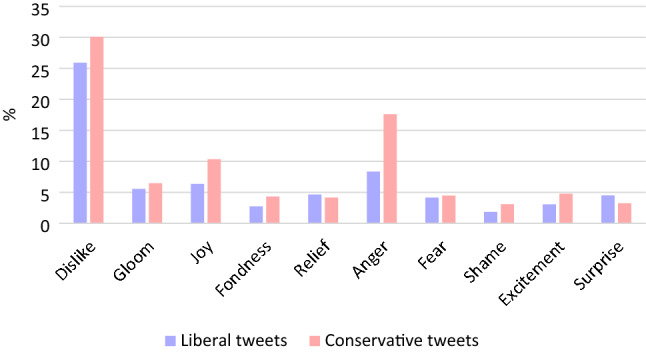
Distributions of emotion words.

Next, we examine the emotion words in tweets, because emotion expressed on social media is contagious, influencing subsequent posts and sharing by readers^31^. For example, Vosoughi et al.^32^ studied the propagation of fake news on Twitter and found that misinformation was more likely to spread when tweets contained emotional content that inspired “surprise” and “disgust”. On the other hand, content expressing sadness, anticipation, and trust promoted the spread of accurate information. Based on these findings, Fig. 4 shows the distributions of emotion words in tweets belonging to the conservative and liberal clusters. To extract emotion words, we used ML-Ask by Ptaszynski et al.^33^, a standard corpus of sentiment words in analyses of the Japanese language.

Figure 4 shows that, in general, the conservative cluster uses emotion words more frequently than the liberal cluster. Statistically significant differences were found in “dislike”, “joy”, “fondness”, “anger”, “shame”, and “excitement”, all of which were more frequent in the conservative cluster. In light of the findings by Vosoughi et al.^32^, the frequent expression of the “dislike” sentiment in conservative clusters may increase the reach of these tweets. However, there was no difference in “surprise”, which Vosoughi et al.^32^ found effective in spreading fake news. This may be due to the qualitative difference between the fake news studied by Vosoughi et al.^32^ and the tweets about Abe in this study, which were not necessarily fake news. Overall, although some emotions were found to be more frequently expressed in conservative tweets than in liberal tweets (e.g., “dislike”), the difference was not sufficiently consistent to explain the liberal–conservative asymmetry in reaching the middle.

Finally, we test the hypothesis that the linguistic expressions used in the conservative and liberal clusters’ partisan messages are different. The use of words is an essential aspect of social identity^34^. Therefore, a message with significantly different words from one’s usual linguistic expressions would be considered to be from a social, political, or cultural out-group, which would reduce the probability of acceptance. In other words, just as the use of partisan hashtags may alienate noncore accounts, moderate users will shun a tweet as coming from a politically extreme out-group if too many partisan expressions are used. More specifically, the word choice of the liberal cluster may diverge further from that of noncore accounts than the conservative cluster, which may make it harder for average users to empathize with them, reducing the liberal cluster’s reach.

**Figure 5:**
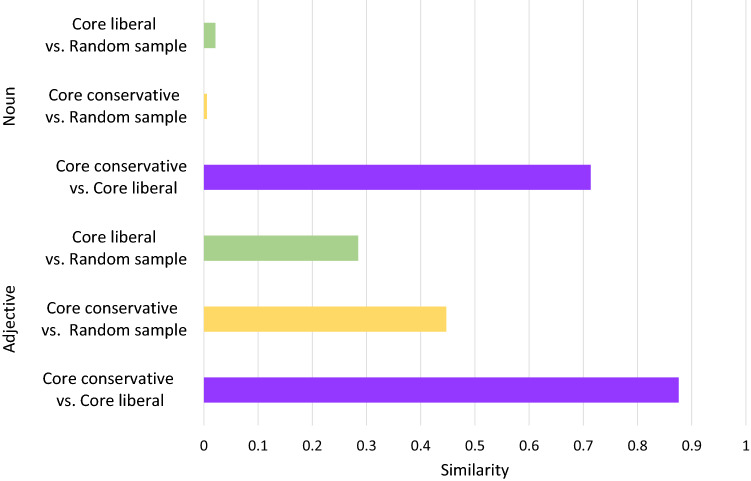
Similarities of linguistic expressions between liberal, conservative, and randomly sampled users.

To probe this hypothesis, we compared the linguistic expressions of conservative, liberal, and randomly sampled users. Random users are those randomly extracted from the stream data, so they are not necessarily those who retweeted the tweet about Prime Minister Abe. We collected the last 200 tweets of the 5000 core accounts with the most posts in each group and examined their linguistic expressions. The words were divided into two groups—nouns and adjectives—and the similarities between the three user groups were compared. The results are shown in Fig. 5. For both nouns and adjectives, there were more similarities between conservative and liberal core users than between the randomly sampled users and either the conservative or liberal users. This result indicates that the strongly ideological conservative and liberal users tend to use similar linguistic expressions. For nouns, the similarity between liberal and conservative users is strong because both groups tend to tweet about politics, but this topic is rarely shared with the random sample, which corroborates the view that average users are largely indifferent to politics. On the other hand, in the use of adjectives, the similarity between liberal and conservative users is highest, but conservative users are more similar to the random sample. Given that random users are likely to be less partisan and more moderate than core liberal or conservative users, liberal users use linguistic expressions that differ further from those used by moderate users, which may restrict the reach of liberal tweets to moderate users.

## Discussion

Examining the penetration of partisan messages to moderate users is imperative to understand the political consequences of social media use. Strongly partisan users have been shown to be embedded in closed and coherent clusters, but little is known how their messages reach beyond cluster borders to moderate users. Focusing on the Japanese Twitterverse, we demonstrated that conservative messages are more effectively conveyed to moderate users than liberal messages. The network analysis indicated that the conservative cluster is structurally better connected to moderate users than the liberal cluster. While the two major partisan clusters’ internal structures were similar, the conservative cluster reciprocated follows from moderate accounts at a higher rate than the liberal cluster. In addition, moderate accounts reciprocated the conservative cluster’s follows at a higher rate than they did for the liberal cluster. On the other hand, the analysis of tweet content showed no difference in the frequency of hashtag use between conservatives and liberals, but there were differences in the use of emotion words and linguistic expressions. In particular, emotion words conveying dislike were used more frequently by conservatives, who also used adjectives in a more similar way to moderate users, indicating that conservative tweets are more palatable to less partisan users than liberal tweets. These differences in content may also assist conservative tweets in reaching the middle.

This study has a number of implications. First, our results strongly indicate that Twitter is not an even playing field for conservatives and liberals in Japan. The more effective use of social media by conservatives than by liberals is consistent with the argument of Bennett et al.^19^. These partisan gaps in reach to moderate users could have tangible electoral consequences. Less partisan, moderate individuals tend to have less political knowledge, so they are relatively persuadable through campaigns^35^. In particular, Japan has witnessed weakening links between political parties and electorates since the 1990s, so it has become crucial to gain support from the nonpartisan electorate to win elections^20^. The fact that conservatives have better reach to the persuadable middle suggests that partisan competition in the digital environment may increasingly favor conservatives.

Second, the most urgent task for liberals is to reestablish connections with the middle. The liberal cluster has a more closed network than the conservative cluster and does not effectively reach the moderate users. Liberals who are critical of the Abe administration tend to be in the middle and older age groups^36^. These generational factors may hinder their effective use of social media. For liberal partisan messages to penetrate in the same way as those from conservatives, they will need to go beyond self-enforcing communication within the partisan enclave and actively build networks to persuade and mobilize the middle.

Third, as a more theoretical implication, this study shows the importance of studying retweets. There is already a considerable body of knowledge on the creation of partisan echo chambers by selective friending on social media^37^. However, the degree of ideological fragmentation alone does not reveal differences in reaching the middle. Because there are generally more moderate voters with weak or no partisanship than strongly partisan voters who form echo chambers in social media, it is crucial to analyze the extent to which partisan messages reach moderate voters.

Several limitations remain to be addressed. First, this study could not examine the impact of bots^38^. If a large number of bots were included that automatically retweet partisan messages, the findings of this study might not accurately reflect behavior by humans. However, our ancillary analysis shows that the impact of bots is not significant, if there is any (see SI2 in the Supplementary Information online). We also examined the possibility that many of the core conservative accounts engage in organized activities, particularly astroturfing by J-NSC^38^, a group of online activists who support the conservative incumbent Liberal Democratic Party, but our results suggest that this is unlikely (see SI3 in the Supplementary Information online).

Second, we found that conservatives are better connected to moderate users, but it is not clear from this study whether this is the result of intentional networking by conservative users. Whether this represents a strategic effort to expand reach to moderates is an issue that future research needs to clarify.

Third, since this study limited its analysis to tweets about Prime Minister Abe who is conservative, it is not clear whether conservatives would still have greater reach to moderate users than liberals if the prime minister were a liberal. A similar analysis is theoretically possible for the opposition leaders, but the volume of (re)tweets about the opposition leader is much lower than that about Prime Minister Abe, and thus stable clusters cannot be detected. However, it should be noted that, as mentioned earlier, conservative tweets are less than liberal tweets, and core conservative accounts are also less than core liberal accounts, so it is difficult to make the interpretation that conservatives tweet more or are more likely to be retweeted simply because Prime Minister Abe is a conservative.

Last, this study is a single-country study focusing only on Twitter in Japan. In this regard, cross-national studies are desired because the way conservatives and liberals connect with moderates may differ depending on political institutions and cultures. Nevertheless, given that most previous studies have been limited to North American and European democracies, it is significant to provide knowledge on Japan as an established democracy in East Asia.

## Supplementary Information

Supplementary Information.

## References

[1] Himelboim I, Smith M, Shneiderman B (2013). Tweeting apart: Applying network analysis to detect selective exposure clusters in Twitter. Commun. Methods Meas..

[2] Liang H, Fu KW (2017). Information overload, similarity, and redundancy: Unsubscribing information sources on Twitter. J. Comput. Mediat. Commun..

[3] Bakshy E, Messing S, Adamic LA (2015). Exposure to ideologically diverse news and opinion on facebook. Science.

[4] Himelboim I, McCreery S, Smith M (2013). Birds of a feather tweet together: Integrating network and content analyses to examine cross-ideology exposure on Twitter. J. Comput. Mediat. Commun..

[5] Barberá P (2015). Birds of the same feather tweet together: Bayesian ideal point estimation using Twitter data. Political Anal..

[6] Leyva R (2017). Exploring UK millennials’ social media consumption patterns and participation in elections, activism, and “slacktivism”. Soc. Sci. Comput. Rev..

[7] Karpf D (2012). Online political mobilization from the advocacy group’s perspective: Looking beyond clicktivism. Policy Internet.

[8] Freelon D, Marwick A, Kreiss D (2020). False equivalencies: Online activism from left to right. Science.

[9] Cohn, N. & Quealy, K. How public opinion has moved on Black Lives Matter. *The New York Times* https://www.nytimes.com/interactive/2020/06/10/upshot/black-lives-matter-attitudes.html (2020).

[10] Freelon, D., McIlwain, C. D. and Clark, M. D. Beyond the hashtags: #Ferguson, #Blacklivesmatter, and the online struggle for offline justice. *Center for Media and Social Impact, American University* https://papers.ssrn.com/sol3/papers.cfm?abstract_id=2747066 (2016).

[11] Zaller JR (1992). The Nature and Origins of Mass Opinion.

[12] Bennett WL, Segerberg A (2012). The logic of connective action: Digital media and the personalization of contentious politics. Inf. Commun. Soc..

[13] Boutyline A, Willer R (2017). The social structure of political echo chambers: Variation in ideological homophily in online networks. Polit. Psychol..

[14] Mannheim K (1936). Ideology and utopia.

[15] Laver M, Benoit K (2005). Estimating party policy positions: Japan in comparative context. Jpn. J. Polit. Sci..

[16] Ito M (2019). The Historical Sociology of the Internet Right: An Underground History of the Heisei Period from 1990s to 2000s.

[17] Kobayashi T, Ikeda K (2007). Socialization of Internet use and its political implications. Political Reality and Social Psychology: Dynamics of Heisei Koizumi Politics.

[18] Nishida R (2018). Politics Armed with Information.

[19] Bennett WL, Segerberg A, Knüpfer CB (2018). The democratic interface: Technology, political organization, and diverging patterns of electoral representation. Inf. Commun. Soc..

[20] Tanaka A, Martin S (2003). The new independent voter and the evolving Japanese party system. Asian Perspect..

[21] Yokoyama T, Kobayashi T (2019). Pitting prime minister cues with party cues in a multiparty system: A survey experiment in Japan. Jpn. J. Polit. Sci..

[22] Uchida K, Sakaki T, Toriumi F (2019). Comparative evaluation of two approaches for retweet clustering: A text-based method and graph-based method. Web Intelligence.

[23] Bae Y, Ryu PM, Kim H (2014). Predicting the lifespan and retweet times of tweets based on multiple feature analysis. ETRI J..

[24] Blondel VD, Guillaume JL, Lambiotte R, Lefebvre E (2008). Fast unfolding of communities in large networks. J. Stat. Mech-theory. E..

[25] Clauset A, Newman ME, Moore C (2004). Finding community structure in very large networks. Phys. Rev. E..

[26] Pons, P. & Latapy, M. Computing communities in large networks using random walks. In *International Symposium on Computer and Information Sciences*. ISCIS2005, Istanbul, October 26–28. pp. 284–293. (2005).

[27] Wakita, K. & Tsurumi, T. Finding community structure in mega-scale social networks. In *Proceedings of the 16th International Conference on World Wide Web*. WWW2007, Banff, May 8–12. pp. 1275–1276. (2007).

[28] Martín Y, Li Z, Cutter SL (2017). Leveraging Twitter to gauge evacuation compliance: Spatiotemporal analysis of Hurricane Matthew. PLoS ONE.

[29] Borge-Holthoefer, J., Magdy, W., Darwish, K., & Weber, I. Content and network dynamics behind Egyptian political polarization on Twitter. In *Proceedings of the 18th ACM Conference on Computer Supported Cooperative Work & Social Computing*. CSCW15, Vancouver, March 14–18. pp. 700–711. (2015).

[30] Xu, B., Huang, Y. & Contractor, N. Exploring Twitter networks in parallel computing environments. In *Proceedings of the Conference on Extreme Science and Engineering Discovery Environment: Gateway to Discovery.* XSEDE13, San Diego, July 22–25. pp. 20. (2013).

[31] Kramer AD, Guillory JE, Hancock JT (2014). Experimental evidence of massive-scale emotional contagion through social networks. Proc. Natl. Acad. Sci. U. S. A..

[32] Vosoughi S, Roy D, Aral S (2018). The spread of true and false news online. Science.

[33] Ptaszynski M, Rzepka R, Araki K, Momouchi Y (2014). Automatically annotating a five-billion-word corpus of Japanese blogs for sentiment and affect analysis. Comput. Speech Lang..

[34] Eastman CM (1985). Establishing social identity through language use. J. Lang. Soc. Psychol..

[35] Prior M (2007). Post-Broadcast Democracy: How Media Choice Increases Inequality in Political Involvement and Polarizes Elections.

[36] Asahi Shumbun. January 2021 Regular RDD Survey. https://digital.asahi.com/politics/yoron/download/202101.pdf.html (2021).

[37] Colleoni E, Rozza A, Arvidsson A (2014). Echo chamber or public sphere? Predicting political orientation and measuring political homophily in Twitter using big data. J. Commun..

[38] Schäfer F, Evert S, Heinrich P (2017). Japan's 2014 general election: Political bots, right-wing internet activism, and prime minister Shinzō Abe's hidden nationalist agenda. Big Data..

