# Supplementary material for: Japanese conservative messages propagate to moderate users better than their liberal counterparts on Twitter
Source: Sci Rep. 2021 Oct 4;11:19224. doi: 10.1038/s41598-021-98349-2 (PMC8488035; doi:10.1038/s41598-021-98349-2)
Supplement: Supplementary file 1 — Supplementary Information. [file 41598_2021_98349_MOESM1_ESM.docx]

Supporting Information

**SI1. Sample tweets of the conservative and liberal tweets**

**Conservative tweets**

“Prime Minister Abe, thank you very much for your long years of hard work. I remember that my grandfather Noboru Takeshita and Mr. Shintaro Abe were good friends, and my grandfather always regretted that he could not make Mr. Abe the Prime Minister. You became Prime Minister twice and had the longest consecutive tenure in history. You were a great Prime Minister. Thank you very much.”

“Old media trying to link Prime Minister Shinzo Abe’s health condition to criticism of the administration, I will never forget your behavior. I will never forget your behavior. Stop underestimating the Japanese people.”

“The opposition media even brought up the story of Abe’s time as secretary-general 35 years ago in an attempt to slam him. But in fact, it turned out to be a fabrication by TV Tokyo. The fact that former Foreign Minister Shintaro Abe was accompanied by the chairman of Japan Life is an outright lie, and the media’s despicable tactics are unbelievable. It’s a lie. This is a crime!”

**Liberal tweets**

“#Step down, Abe! To be honest, if you think Shinzo Abe and Taro Aso are the worst prime minister and deputy prime minister in the postwar era, please RT and like this tweet.”

“Shinzo Abe’s speeches are all the same every year, not only for Hiroshima and Nagasaki “Atomic Bomb Days,” but also for Okinawa Memorial Day on June 23 and for the “End of the War Day” on August 15. It’s easy to see if you search the entire text on the Internet. It is unprecedented for a prime minister to show such disregard for the victims of war.”

“As I watched the parliamentary questioning, I was reminded that Prime Minister Abe did not answer the opposition members’ questions properly, but only read the prepared text. Eventually, the time ran out, and it was over. Some people say that the opposition parties are not pursuing the issue enough, but this cannot be Q&A. NHK, on the other hand, cut out only the part of Abe’s answer that was convenient for him and reported it. The whole thing is corrupt.”

**SI2. Ancillary analysis of the potential impact of bots**

First, we checked whether there were any accounts that were specifically created to spread the partisan tweets. As a result, only 1.3% to 3.5% of the accounts had posted fewer than 10 tweets when they posted the partisan tweets in our data, which is smaller than the 7.6% from the stream data. If bots tend to be newly created to spread specific partisan messages, these results indicate they are less likely to be included in our data compared with the stream data. Next, we checked where the tweets were posted from. The ratios of partisan tweets from either iPhones or Android phones were 81.8% and 82.0% for the core liberal and core conservative accounts, respectively, and these numbers did not differ from the stream data. Of course, it is possible to disguise the source of bot postings as iPhone or Android phone by using advanced technology, but the idea that these accounts are responsible for the majority of bot postings is not very realistic. In addition, the percentage of users who use twittbot.net, a bot creation application, or other platforms, is rather smaller than that of streams, suggesting that there is little use of bots.

**SI3. Ancillary analysis of the potential astroturfing by J-NSC**

Considering the possibility that the dissemination of messages by core conservative accounts is an organized activity, we analyzed the presence of J-NSC among core conservative accounts. J-NSC is a group of Internet users who support the conservative Liberal Democratic Party (LDP). It has been pointed out that they support the LDP and systematically attack the liberal opposition parties on different online platforms ^[1]^. First, we identified 281 accounts in our dataset that included “J-NSC” or “LDP Net Supporters Club (自民党ネットサポーターズクラブ)” in their account profiles. Of these accounts, 106 (0.1%) were included in the 88,709 core conservative accounts. Next, @j_nsc_jp, which appears to be the official account of J-NSC, had 10,077 followers. However, this account has been dormant since 2017. Of these accounts, 1,058 were included in the core conservative accounts (1.2%). Thus, as far as we can analyze from the observable cues of profile information and following @j_nsc_jp, the presence of J-NSC in core conservative accounts is not notable. Of course, we cannot exclude the possibility that there are members who are active without explicitly indicating their affiliation with J-NSC, but many of the core conservative accounts are relatively unlikely to be organized astroturfing.

1. Schäfer, F., Evert, S. & Heinrich, P. Japan’s 2014 general election: Political bots, right-wing internet activism, and prime minister Shinzō Abe’s hidden nationalist agenda. *Big Data*. **5**, 294–309 (2017).
